# Original Method of Making a Matrix for Inlays

**Published:** 1904-07

**Authors:** Charles Channing Allen

**Affiliations:** Kansas City, Mo.


### ORIGINAL METHOD OF MAKING A MATRIX FOR INLAYS.

By Charles Channing Allen, D. D. S., Kansas City, Mo.

(Written for *The American Journal of Dental Science.*)

No baked inlay can be successful or satisfactory unless the matrix in which it is made be good. The making of a matrix has in the past proved one of the most difficult processes connected with inlay work. This of course obtains because of the extreme delicacy of the foil used for this purpose, whether it be platinum or gold.

It is not only difficult to form the matrix of this material perfectly but also difficult to remove it from the cavity and invest it, if investment be desired, without distortion. If the matrix could be filled with some more or less resisting material in the mouth, this material being swaged by pressure into the cavity, thereby making the foil confirm perfectly to the cavity and all its edges and then remove bodily for investment, the process would be simplified.

Many substances have been suggested for this purpose but the difficulty has always been to get rid of them after they have served their end. This difficulty has been found to be such that the benefit derived from their use has been largely minimized.

After considering and using all the materials that have been brought to my attention and that I could think of, it occurred to me to use gum camphor, which I find almost ideally fills the bill.

After the cavity has been outlined on the foil by the use of cotton, spunk, or other similar material, a small piece of gum camphor may be placed over the cavity and firmly pressed into position by the use of proper burnishers and instruments and it will be found to swage the foil perfectly to the cavity walls. This may be burnished down very much as though one intended to make a permanent filling of the gum camphor, you can thereby get a perfect edge all around your cavity.

After the camphor has been worked into position in this manner the matrix, camphor, and all may be removed in the usual way and invested as one may desire. If you use alcohol in your investment, simply set it afire and by the time the alcohol has burned up, the camphor will be found to have entirely disappeared, leaving no trace whatever of its former tenancy. This is, of course, the very thing desired.

If it is not desired to invest the matrix, simply set the camphor afire with a match or an alcohol flame and it will disappear as before. This method is especially useful where one desires to use pins, staples or other retaining devices which must be held in certain absolutely relative positions. This can be done with camphor and no question of error may arise.

In contour work and in shallow cavities some times the camphor will be found a little hard to work but skill in manipulation will very largely overcome this defect and the operator will find much satisfaction in the use of this material.

There is nothing about the camphor that is in any way offensive to the patient or that makes it undesirable to use about the mouth.

---

### PORCELAIN INLAYS.

By H. E. Phillips, D. D. S., Canton, O.

(Written for *The American Journal of Dental Science.*)

